# Pseudorabies Virus UL24 Abrogates Tumor Necrosis Factor Alpha-Induced NF-κB Activation by Degrading P65

**DOI:** 10.3390/v12010051

**Published:** 2020-01-02

**Authors:** Tong-Yun Wang, Yue-Lin Yang, Cong Feng, Ming-Xia Sun, Jin-Mei Peng, Zhi-Jun Tian, Yan-Dong Tang, Xue-Hui Cai

**Affiliations:** 1 State Key Laboratory of Veterinary Biotechnology, Harbin Veterinary Research Institute of Chinese Academy of Agricultural Sciences, Harbin 150001, China; sdndwty@163.com (T.-Y.W.); 15663830363@163.com (Y.-L.Y.); qzsmx122@163.com (M.-X.S.); peng_jinmei@126.com (J.-M.P.); 2 Guangdong Key Laboratory of Laboratory Animals, Guangdong Laboratory Animals Monitoring Institute, Guangzhou 510000, China; congfeng521@126.com

**Keywords:** TNF-α, NF-κB, pseudorabies virus, UL24, P65

## Abstract

The transcription factor NF-κB plays a critical role in diverse biological processes. The NF-κB pathway can be activated by incoming pathogens and then stimulates both innate and adaptive immunity. However, many viruses have evolved corresponding strategies to balance NF-κB activation to benefit their replication. Pseudorabies virus (PRV) is an economically important pathogen that belongs to the alphaherpesvirus group. There is little information about PRV infection and NF-κB regulation. This study demonstrates for the first time that the UL24 protein could abrogate tumor necrosis factor alpha (TNF-α)-mediated NF-κB activation. An overexpression assay indicated that UL24 inhibits this pathway at or downstream of P65. Furthermore, co-immunoprecipitation analysis demonstrated that UL24 selectively interacts with P65. We demonstrated that UL24 could significantly degrade P65 by the proteasome pathway. For the first time, PRV UL24 was shown to play an important role in NF-κB evasion during PRV infection. This study expands our understanding that PRV can utilize its encoded protein UL24 to evade NF-κB signaling.

## 1. Introduction

Nuclear factor κB (NF-κB) plays important roles in diverse biological processes by regulating the expression of a large number of target genes that are involved in the immune and inflammatory response, cell proliferation and survival [1,2,3,4]. NF-κB always consists of dimeric transcription factors that belong to the Rel family; in mammalian cells, the predominant form of NF-κB is a heterodimer composed of p50 and RelA (P65) subunits [5]. Without stimulation, inactive NF-κB is sequestered in the cytoplasm by IκB inhibitory proteins, usually IκBα. When cells are stimulated by stress, inflammatory cytokines, or bacterial or viral pathogens, IκBα is activated by an upstream kinase and degraded by the proteasome pathway, and NF-κB dimers are released and further translocate to the nucleus to activate the transcription of a variety of genes [4].

Activation of NF-κB is a rapid cellular event that occurs within minutes after virus invasion. NF-κB does not require novel viral protein synthesis and is able to affect many critical steps in the host cell, making NF-κB an attractive target for an invading virus [4]. An increasing number of studies have indicated that triggering NF-κB activation is a double-edged sword, particularly during viral infection. On the one hand, NF-κB activation blocks apoptosis and prolongs survival of the infected cell to gain enough time for replication [6]. On the other hand, NF-κB plays a pivotal role in eliciting innate and adaptive immunity and inflammation responses, which in turn limits viral replication [2,3,5,7]. Successful viral replication requires balancing NF-κB activation to evade the host immune response. Several viruses have evolved distinct strategies to modulate the NF-κB pathway [8]. A good example is herpes simplex virus 1 (HSV-1), which encodes several gene products that enable the virus to block NF-κB responses [9,10,11,12,13,14,15].

Pseudorabies virus (PRV) is a swine alphaherpesvirus that causes Aujeszky’s disease in pigs [16,17]. It is a neglected zoonotic pathogen in humans [18], and recent studies revealed that PRV may be a potential threat to humans [19,20]. These studies showed that pseudorabies virus might infect humans after direct contact with contaminants and that the patients presented with high-grade fever, headache and tonic–clonic seizure as well as coma [19,20]. Therefore, in the current study we used human cells (HEK293) to explore whether PRV could block NF-κB responses. Recent studies demonstrated that PRV could resemble HSV-1 in many aspects; despite substantial divergence in their sequences, PRV serves as a useful model system for studying alphaherpesvirus biology [21,22,23,24,25,26]. Whether PRV encodes protein(s) that negatively regulate the NF-κB pathway, as HSV-1 does, is unclear. In this report, we show that UL24 impairs TNF-α-mediated NF-κB activation and that, mechanistically, UL24 interacts with P65 and further mediates P65 degradation in the proteasome pathway. Our results were the first to show that UL24 is a potent NF-κB modulator during PRV infection.

## 2. Materials and Methods 

### 2.1. Virus, cells and reagents

The PRV HeN1 strain (GenBank accession number: KP098534.1) was isolated in our laboratory, and the properties of the HeN1 strain have been described previously [27,28,29]. Vero cells, HEK293T cells, and HeLa cells were cultured in Dulbecco Modified Eagle Medium (DMEM) (Gibco) with 10% fetal bovine serum (HyClone). MG132 and CQ were purchased from Sigma.

### 2.2. Transfection and dual-luciferase reporter (DLR) assay

HEK293T cells or HeLa cells were seeded in 96-well plates. At approximately 90% confluence, the cells were transfected with NF-κB-Luc reporter plasmids with or without an expression plasmid, as indicated by the Polyjet DNA transfection reagent (Roche, Basel, Switzerland), following the manufacturer’s protocol. Renilla luciferase activity was used as an internal control for the normalization of luciferase values obtained from cells. At 24 h post transfection, the cells were lysed, and luciferase assays were performed using a Dual-Luciferase Reporter (DLR) assay (Promega, Madison, USA) according to the manufacturer’s instructions.

### 2.3. PRV UL24-Null Mutant Construction

Target sequences were synthesized and cloned into the PX330 vector, as previously reported [29,30,31,32,33,34]. UL24 was knocked out by two sgRNAs, and the targeting sites were 5′-GCGGCACCGGCAAGAGCACCA-3′ and 5′-GTGCGCCTTCACGTCGGAGAT-3′, respectively. The genomic DNA of PRV HeN1 was extracted as previously described [28]. Vero cells were plated into a 6-well plate when the cells were approximately 90% confluent, and 3 µg of PRV HeN1 genomic DNA was co-transfected with 1 µg of each CRISPR construct together with 10 µL of X-tremeGENE HP DNA Transfection Reagent (Roche, Basel, Switzerland). After 72 h, the cells were collected and subjected to three freeze–thaw cycles. The virus was purified via plaque purification, and purified clones were randomly picked. For further confirmation of UL24 knockout, viral DNA derived from different clones was extracted and identified by PCR. The primers used for UL24 knockout identification were as follows: UL24-F, 5′-atgcgcatcccggcgcg-3′; and UL24-R, 5′-tcaccgccgcggcccgg-3′.

### 2.4. Co-Immunoprecipitation and WB Analysis

HEK293T cells (6 × 10^6^) were co-transfected with the indicated expression plasmids carrying a Flag or HA tag. Thirty-six hours after transfection, the sample was lysed on ice with 500 μL of lysis buffer. A 0.4-mL lysate was incubated with 30 µL of beads conjugated with anti-Flag MAb overnight at 4 °C. The beads were washed six times with 1 mL of PBS at 1500× *g* for 2 min and then subjected to western blot (WB) analysis. The Western protocol was the same as one previously described [31]. Briefly, cell lysates and immunoprecipitated proteins were separated in denaturing 12% polyacrylamide gels and transferred to polyvinylidene difluoride (PVDF) membranes. Then, after blocking with 5% nonfat milk in phosphate buffer saline (PBS) and washing with Tris-Buffered Saline Tween-20 (TBST) three times, the membranes were incubated for 2 h at room temperature with the following primary antibodies diluted as indicated: anti-Flag (1:2000; Sigma) or anti-HA (1:2500; Sigma), followed by incubation with the appropriate secondary antibody, goat anti-mouse IgG, for one hour.

### 2.5. Preparation of UL24 Protein

The UL24 gene was first cloned into the PET-30 vector, and the recombinant strain was inoculated at a 1% (*v*/*v*) ratio into 100 mL of fresh Luria Broth (LB) (Amp 50 µg/mL) and cultured for 3–4 h at 37 °C. A final concentration of 1.0 mmol/L IPTG was used to induce the expression of the UL24 protein. Subsequently, the cell lysate was centrifuged at 12,000 r/min for 10 min, and the inclusion bodies were suspended in binding buffer containing 6 mmol/L urea and incubated at 4 °C for 12 h. Then, centrifuging 12,000 r/min for 15 min, the target protein containing the supernatant was analyzed and purified by Sodium dodecylsulphate polyacrylamide gel electrophoresis (SDS-PAGE).

### 2.6. Preparation of Anti-UL24 Rabbit Polyclonal Antibody

The purified UL24 protein was used to immunize New Zealand white rabbits to obtain polyclonal antibodies. The immunization procedure was as follows. The purified UL24 protein (1 mg/mL) was mixed with Freund’s adjuvant (complete). Subsequently, an antigen–adjuvant emulsion was subcutaneously injected. Two weeks, four weeks, and six weeks after the first immunization, 1 mg of the purified UL24 protein mixed with Freund’s adjuvant (incomplete) was subcutaneously injected. The post-immunization serum was collected at seven weeks. Polyclonal antibodies against PRV UL24 were confirmed by Western blotting.

### 2.7. Semiquantitative PCR

HeLa cells were transfected with 2 μg of UL24 plasmid or an empty vector. At 24 h post transfection, the cells were mock treated or treated with TNF-α (10 ng/μL) for 8 h. Total RNA was extracted from HeLa cells with Trizol reagent (Sangon, Shanghai, China) according to the manufacturer’s instructions. Samples were digested with DNase I and subjected to reverse transcription. The cDNA was used for semi-quantitative PCR to investigate the accumulation of human IL-6 and IL-8 mRNA and UL24 as previously described. The primers for each gene of interest were as follows: GAPDH, 5′-TGACCTCAACTACATGGTTTACATGT-3′ and 5′-AGGGATCTCGCTCCTGGAA-3′; IL-6, 5′-GGATTCAATGAGGAGACTTGCC-3′ and 5′-ACAGCTCTGGCTTGTTCCTCAC-3′; IL-8, 5′-CAACACAGAAATTATTGTAAAGCTTTCT-3′ and 5′-GAATTCTCAGCCCTCTTCAAAAA-3′; and UL24-F, 5′-ATGCGCATCCCGGCGCG-3′ and 5′-TCACCGCCGCGGCCCGG-3′.

### 2.8. Replication Kinetics of Wild Type (WT) PRV or UL24 Knockout PRV by qPCR

The HEK293 cells were inoculated with WT PRV or UL24 knockout PRV at a multiple of infection (MOI) of 0.1. The virus was collected at 4, 8, 16, and 24 h post infection. qPCR was used to quantify the copy numbers of viral DNA. This method was based on the gB gene of PRV (forward primer: 5′-acggcacgggcgtgatc-3′, reverse primer 5′-actcgcggtcctcgagca-3′, TaqMan probe FAM-ctcgcgcgacctcatcgagccctgcac-MGB.

### 2.9. Immunofluorescence Assay

HEK293T cells were transfected with 2 μg of UL24 plasmid or an empty vector. At 24 h post transfection, cells were fixed in 4% paraformaldehyde for 1 h, washed three times with PBS and then permeabilized with 0.2% Triton X-100 for 1 h. After washing with PBS three times, cells were blocked with 1% BSA for 2 h and then incubated for 1 h at room temperature with antibody specific for HA and P65. For nuclear visualization, cells were stained with DAPI (Sigma, St Louis, USA).

### 2.10. P65 Polyubiquitination Assay

HEK293T cells were co-transfected with the HA-tagged UL24, FLAG-tagged p65, and HA-tagged Ub expression vector at a 1:1:1 ratio using the calcium phosphate method. Protein was extracted 30 h post transfection. The P65-ubiquitin complexes were immunoprecipitated using anti-FLAG antibody M2-conjugated beads (A2220, Sigma) and immunoblotted with anti-HA antibody to detect ubiquitinated proteins.

### 2.11. Statistical Analysis

All the graphs and relevant statistical tests used in the work were created by GraphPad Prism version 6.00 (La Jolla, CA, USA). Statistical significance between two groups was analyzed by two-tailed unpaired Student’s *t*-test.

## 3. Results

### 3.1. PRV UL24 Dampens TNF-α-Mediated NF-κB Activation

**Figure 1:**
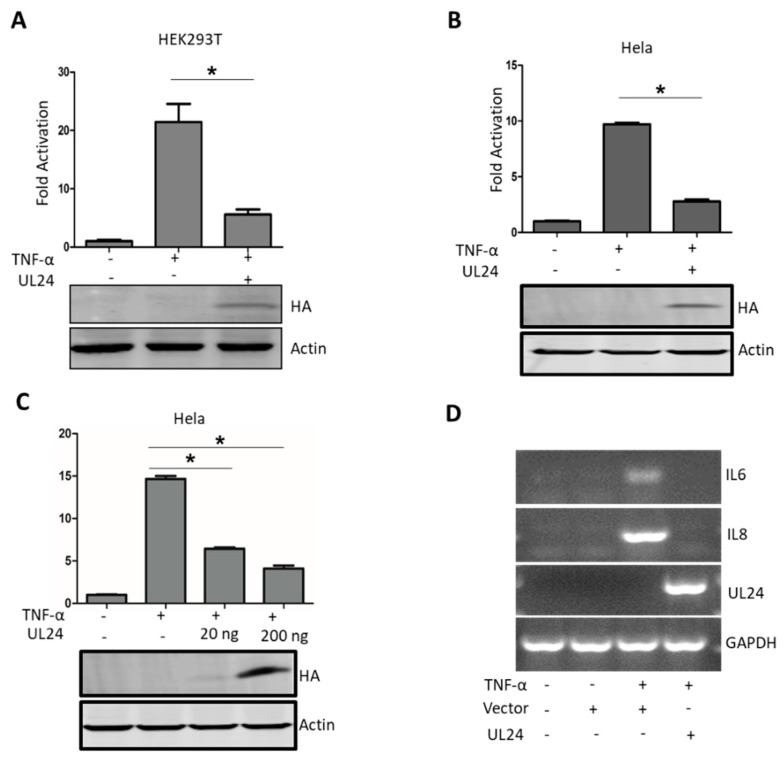
Pseudorabies virus (PRV) UL24 dampens TNF-α-mediated NF-κB activation. (**A**) HEK293T cells and (**B**) HeLa cells were co-transfected with an NF-κB-Luc reporter plasmid, the pRL-TK plasmid (transfection control), along with a UL24 plasmid or an empty vector. Luciferase activity was measured at 24 h post transfection, and fold activation was determined compared to that of the empty vector (fold activation of the empty vector was set as 1). The expression of UL24 was analyzed by WB using an anti-HA MAb. (**C**) HeLa cells were co-transfected with 200 ng of p65 plasmid and an empty vector or an increasing amount of the UL24 plasmid (0, 20 ng, and 200 ng); the cells were lysed at 48 h post transfection, and luciferase activity was measured. The expression of UL24 was analyzed by WB using an anti-HA Mab. (**D**) HeLa cells were transfected with 2 μg of UL24 plasmid or an empty vector. At 24 h post transfection, the cells were mock treated or treated with TNF-α (10 ng/μL) for 8 h. Total RNA was extracted and digested with DNase I and further subjected to reverse transcription. The cDNA was used for semi-quantitative PCR to investigate the accumulation of human IL-6 and IL-8 mRNA and UL24. The data represent results from one of three independent experiments. Error bars represent standard deviations of data from three replicates of one independent experiment. * stands for *p* < 0.05.

TNF-α is a multifunctional pro-inflammatory cytokine involved in protecting the host from pathogen infections by induction and regulation of host innate and adaptive immune responses [35]. TNF-α regulates host innate and adaptive immune responses mainly by the NF-κB pathway; it first binds to its receptor and subsequently recruits corresponding adaptors, finally activating NF-κB [36]. In this study, we used TNF-α as a stimulator to test whether UL24 plays a role in TNF-α-mediated NF-κB activation. As shown in Figure 1A, TNF-α significantly promoted NF-κB reporter gene expression, and UL24 significantly attenuated TNF-α-mediated NF-κB activation in HEK293T cells (Figure 1A). We next evaluated whether UL24 blocks NF-κB activation in HeLa cells. We got a similar result as in HEK293T cells (Figure 1B). Furthermore, we found that this attenuation was dose dependent (Figure 1C). IL-6 and IL-8 mRNA were downstream of NF-κB, so we next evaluated whether UL24 influences the expression of IL-6 and IL-8. The result demonstrated that UL24 expression significantly downregulated IL-6 and IL-8 (Figure 1D). These data indicated that UL24 overexpression significantly reduced TNF-α-mediated NF-κB activation.

### 3.2. A UL24 Knockout Virus Enhanced NF-κB Activation Compared to That Induced by Wild-Type Virus

**Figure 2:**
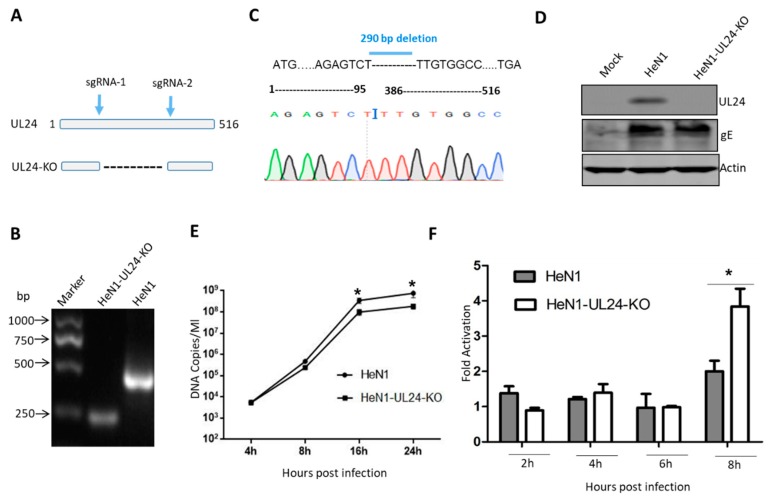
Deletion of UL24 promotes NF-κB activation. (**A**) Diagram of the PRV UL24 gene, with the sgRNA1 and sgRNA2 incision positions. (**B**) The UL24 knockout virus was identified by PCR. Several clones were randomly chosen and cultured in Vero cells, and the virus genome was extracted and identified by PCR with the primers. (**C**) UL24 knockout was confirmed by DNA sequencing; 290 bases were deleted from 96~385 bp of the UL24 gene. The deletion region is shown as a dotted line. (**D**) UL24 knockout was confirmed by Western blot. (**E**) The HEK293 cells were inoculated with WT PRV or UL24 knockout PRV at a multiple of infection (MOI) of 0.1. The virus was collected at 4, 8, 16, and 24 h post infection. qPCR was used to quantify the copy numbers of viral DNA. (**F**) HEK293T cells were transfected with an NF-κB-Luc reporter plasmid. Twelve hours later, the cells were infected with HeN1 PRV or UL24 knockout PRV (MOI = 10), and luciferase activity was measured at 2 h, 4 h, 6 h, and 8 h post infection. The experiments were performed three times, and a representative result is shown. * indicates *p* < 0.05.

To determine whether this negative regulation also existed in PRV infection, we generated a UL24 knockout PRV. First, the knockout strategy is shown in Figure 2A, and two sgRNAs were designed as indicated. If UL24 was knocked out as expected, it would produce a shorter PCR product than the wild-type virus (Figure 2A). PCR identification, DNA sequencing, and Western blot showed that UL24 was successfully knocked out, as expected (Figure 2B–D). We next evaluated whether UL24 knockout influences PRV replication. The result indicated that the UL24-KO virus manifested no significant difference from the WT virus at the early stage; however, the UL24-KO virus replicated more slowly than did the WT virus at the late stage (Figure 2E).To test the role of UL24 in the negative regulation of NF-κB in PRV infection, we used PRV HeN1 and PRV-UL24-KO to infect HEK293T cells at a multiplicity of infection (MOI) of 10. PRV-UL24-KO virus activated NF-κB reporter gene expression similarly to PRV HeN1 at 2, 4, and 6 h (Figure 2D), but it activated NF-κB reporter gene expression at 8 h significantly more than PRV HeN1 (Figure 2D). This result suggested that UL24 was critical for attenuating NF-κB activation.

### 3.3. UL24 Inhibits the NF-κB Signaling Pathway at or Downstream of P65

**Figure 3:**
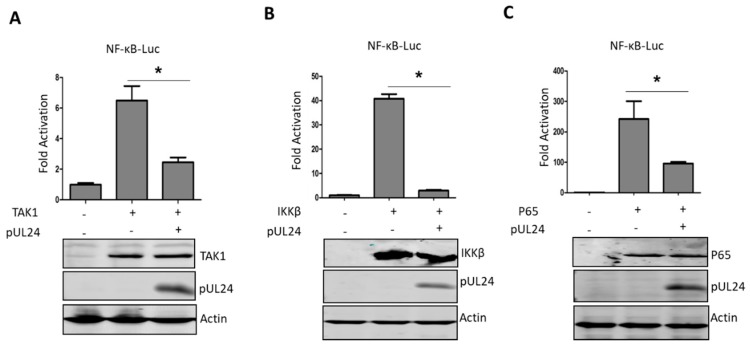
UL24 inhibits the TNF-α-mediated NF-κB signaling pathway via p65. (**A**) HEK293T cells were transfected with 200 ng of the NF-κB-Luc promoter reporter plasmid, the Renilla luciferase plasmid pRL-TK (50 ng), and 200 ng of TAK1 (**A**), IKKβ (**B**), or p65 (**C**) expression plasmids along with pCAGGS-HA empty vector or pCAGGS -UL24 (200 ng), and luciferase activity was measured at 24 h post transfection. Cell lysates were analyzed by WB with Flag-specific antibodies to detect the expression of TAK1-Flag, IKKβ-Flag, and p65-Flag, and HA-specific antibodies were used to detect UL24 expression. The data represent results from one of three independent experiments. Error bars represent standard deviations of data from three replicates of one independent experiment. * stands for *p* < 0.05.

Next, we further explored which step(s) in the NF-κB signaling pathway was/were abrogated by UL24. TNF-α activates NF-κB by first binding to its receptor, resulting in recruitment of the adaptor protein TNF receptor death domain (TRADD), TNFR-associated factor 2 (TRAF2), and receptor-interacting protein 1 (RIP1), and then activates TGF-activated kinase 1 (TAK1) to further activate NF-κB [36]. TAK1 is one of the most important regulatory components in the NF-κB signaling pathway [37], so we first determined whether the UL24 targeting site was upstream or downstream of TAK1. As shown in Figure 3A, UL24 significantly inhibited TAK1-induced NF-κB activation. Next, we further tested the targeting site at the IκB kinase beta (IKKβ) or P65 level. The results demonstrated that UL24 inhibited NF-κB activation induced by IKKβ or P65, indicating that the UL24 targeting site was at or downstream of P65 (Figure 3B,C).

### 3.4. UL24 Degrades P65 in PRV Infection

**Figure 4:**
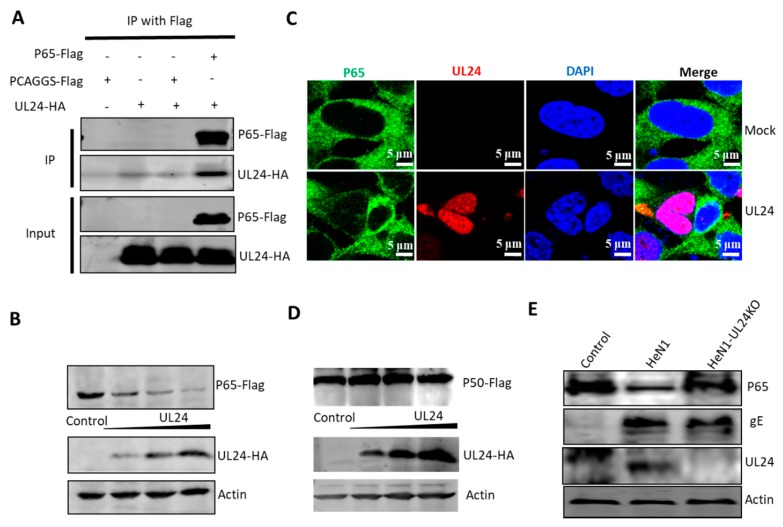
UL24 interacts with p65 and leads to its degradation. (**A**) HEK293T cells were co-transfected with a p65-Flag expression plasmid and a UL24-HA expression plasmid for 48 h. The cells were lysed, and the extracts were processed for immunoprecipitation with an anti-Flag antibody. Precipitated proteins and whole-cell lysates were probed with antibodies against HA and Flag. (**B**) HEK293T cells were co-transfected with increasing amounts of the UL24 plasmid (0, 0.5, 1, and 2 μg) and 2 μg of the p65 plasmid; the cells were lysed at 48 h post transfection, and the expression of p65 was detected by WB. (**C**) HEK293T cells were transfected with 2 μg of UL24-HA expression plasmid. At 48 h post transfection, the P65 status of the cells was detected by immunofluorescence, scale bar was 5 μm. (**D**) HEK293T cells were co-transfected with increasing amounts of the UL24 plasmid (0, 0.5, 1, and 2 μg) and 2 μg of the p50 plasmid; the cells were detected by WB. (**E**) HEK293T cells were infected with wild-type PRV or UL24 knockout PRV at an MOI of 0.01 for 48 h. The cells were lysed, and the extracts were subjected to WB. The experiment was performed three times, and a representative result is shown.

To investigate the mechanism by which UL24 inhibits NF-κB activation, we tested whether there exists a potential interaction between UL24 and p65. UL24-HA and p65-Flag expression plasmids were co-transfected into HEK293T cells and immunoprecipitated with an anti-Flag antibody. The results indicated that P65 efficiently interacted with UL24 (Figure 4A). Next, we further tested whether P65 expression was downregulated by UL24; HEK293T cells were co-transfected with UL24-HA and p65-Flag expression plasmids. Surprisingly, P65 was robustly degraded by UL24 in a dose-dependent manner (Figure 4B). We further tested whether UL24 degraded endogenous p65. By immunofluorescence assay, we found that UL24 mainly located at nucleus, and the P65 level was very low in the cells in the presence of UL24 (Figure 4C). However, when we tested whether UL24 influences P50 expression, we found that UL24 had no influence (Figure 4D). We next tested whether UL24 degraded endogenous p65 in the context of PRV infection. HEK293T cells were infected with 0.1 MOI PRV HeN1 or PRV-UL24-KO, the cells were lysed at 24 h post infection, and endogenous p65 expression was detected by using an anti-P65 antibody. The results demonstrated that endogenous p65 was degraded by PRV HeN1 but not by PRV-UL24-KO, indicating that UL24 could potently degrade P65 in PRV infection (Figure 4C).

### 3.5. UL24 Degrades P65 via the Proteasome Pathway

**Figure 5:**
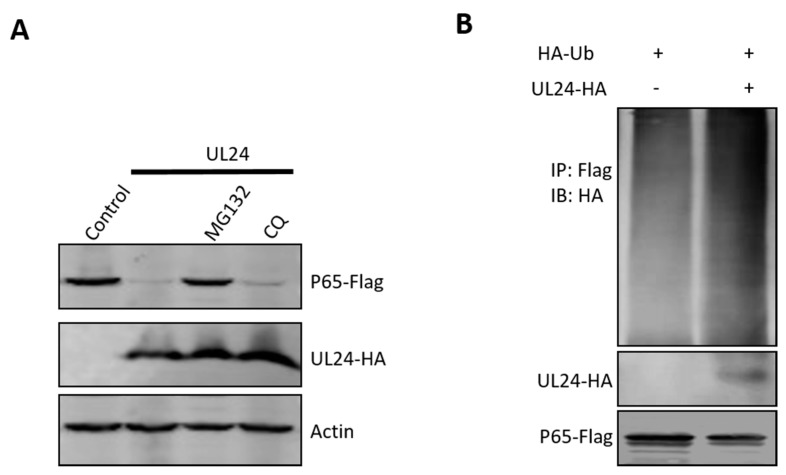
UL24 degrades p65 through the proteasome pathway. (**A**) HEK293T cells were co-transfected with a p65-Flag expression plasmid and a UL24 plasmid or an empty vector. Six hours later, the proteasome inhibitor MG132 (7.5 μM) and the lysosome inhibitor CQ (50 μM) were added. Cell lysates were analyzed by WB. (**B**) HEK293T cells were co-transfected with the HA-tagged UL24, FLAG-tagged p65, and HA-tagged Ub 30 h post transfection. The P65-ubiquitin complexes were immunoprecipitated using anti-FLAG antibody and immunoblotted with anti-HA antibody to detect ubiquitinated proteins.

For intracellular protein degradation, there are two major pathways: the ubiquitin–proteasome-dependent pathway and the lysosome-dependent pathway. To determine if these two pathways are involved in UL24-mediated P65 degradation, MG132 (ubiquitin–proteasome inhibitor) and chloroquine (CQ, lysosome pathway inhibitor) were added to HEK293T cells co-expressing UL24 and P65. The Western blot (WB) results shown in Figure 4 indicate that the ubiquitin–proteasome inhibitor MG132 reversed the expression of P65 and that CQ did not. This result indicated that UL24 degrades P65 via the proteasome pathway. Proteins degraded via the proteasome pathway must be ubiquitinated first. The above data demonstrated that UL24 interacted well with P65; we next tested whether UL24 affects P65 ubiquitination. The ubiquitination assay showed that UL24 could increase P65 polyubiquitination (Figure 5B). 

## 4. Discussion

Previous studies have indicated that PRV has a potent capacity to suppress innate signaling [38,39,40]. However, the mechanism is largely unknown, and only a recent study revealed that the PRV UL50 protein possesses the ability to suppress type I Interferon (IFN) signaling by promoting lysosomal degradation of IFNAR1 [41]. Our study expanded our detailed knowledge of how PRV evades host innate signaling.

The relationship between viral infection and the host NF-κB response is quite complex and has been well discussed in previous works, and some successful viruses, such as HSV-1, have evolved corresponding strategies to maintain the balance of the NF-κB response [2,4,8,10,42]. In the current study, we demonstrated that UL24 of PRV is a key NF-κB regulator in PRV infection, and this activity may be critical for the PRV life cycle. Indeed, the function of the PRV UL24 gene was unclear until our current study, and UL24 of HSV-1 has been reported to block NF-κB activation by hindering nuclear translocation of p65 and p50 [14]. However, our study showed that PRV UL24 was distinct from HSV-1 UL24 and that PRV UL24 terminated NF-κB activation by degrading P65.

Many herpesviruses, such as Epstein–Barr virus (EBV) [43], varicella–zoster virus (VZV) [44,45,46] and HSV-1, encode viral proteins to balance NF-κB activation. HSV-1 is the most well-studied herpesvirus, and it encodes many different viral proteins to counteract host immune responses, as reviewed recently [47,48]. However, the counteraction mechanisms are quite distinct; for example, the protein kinase US3 blocks NF-κB activation by hyperphosphorylating p65 [49], ICP0 works by degrading P50 [10], ICP27 is associated with stabilizing IκBα [9], γ_1_34.5 inhibits p65 phosphorylation [11], VP16 interacts with p65 [13], and UL42 retains p65 and p50 in the cytoplasm [15]. In summary, there are no HSV-1 proteins that block NF-κB activation by degrading p65, as PRV UL24 was shown to do in the current study.

ICP0 of HSV-1 has the ability to degrade P50, and VZV ORF61 degrades IRF3 because ICP0 and ORF61 are E3 ubiquitin ligases [10,50,51,52,53,54,55]. Our study demonstrated that UL24 degrades P65 by the proteasome pathway. Because PRV UL24 is not an E3 ubiquitin ligase, we speculated that UL24 may be an E3 ubiquitin ligase adaptor that can recruit specific E3 ubiquitin ligases to degrade target proteins. This activity may be similar to that of small accessory proteins of some lentiviruses [56]. The APOBEC3 (A3) deoxycytidine deaminases are intracellular restriction factors against HIV-1 [57,58], and HIV-1 viral infectivity factor (Vif) recruits the Cullin5-Ring E3 (Cul5-E3) ubiquitin ligase complex to induce polyubiquitination and degradation of A3 enzymes [59,60]. In addition to the above-summarized lentiviruses, ORF63 of VZV is neither an E3 ligase, but it has the ability to degrade IRF9 by the proteasomal pathway [61]. Therefore, we propose that PRV UL24 is an E3 ubiquitin ligase adaptor, as described above. Some E3 ubiquitin ligases have been reported to be essential for the control of p65 by inducing its degradation, for example, peroxisome proliferator activated receptor-γ (PPARγ), PDLIM2 and COMMD1 [42,62,63,64]. However, which E3 ubiquitin ligase was recruited by UL24 to degrade P65 was unclear in the current study; therefore, the exact mechanism by which UL24 elicits the degradation of p65 still needs to be elucidated in the future.

In conclusion, this report is the first description dissecting the role of UL24 in PRV infection. These data also contribute to a better understanding of the role of PRV-encoded proteins that tightly control innate signaling evasion.
